# Supplementary figures and images for: RANK+TLR2+ myeloid subpopulation converts autoimmune to joint destruction in rheumatoid arthritis
Source: eLife. 2023 May 19;12:e85553. doi: 10.7554/eLife.85553 (PMC10299822; doi:10.7554/eLife.85553)

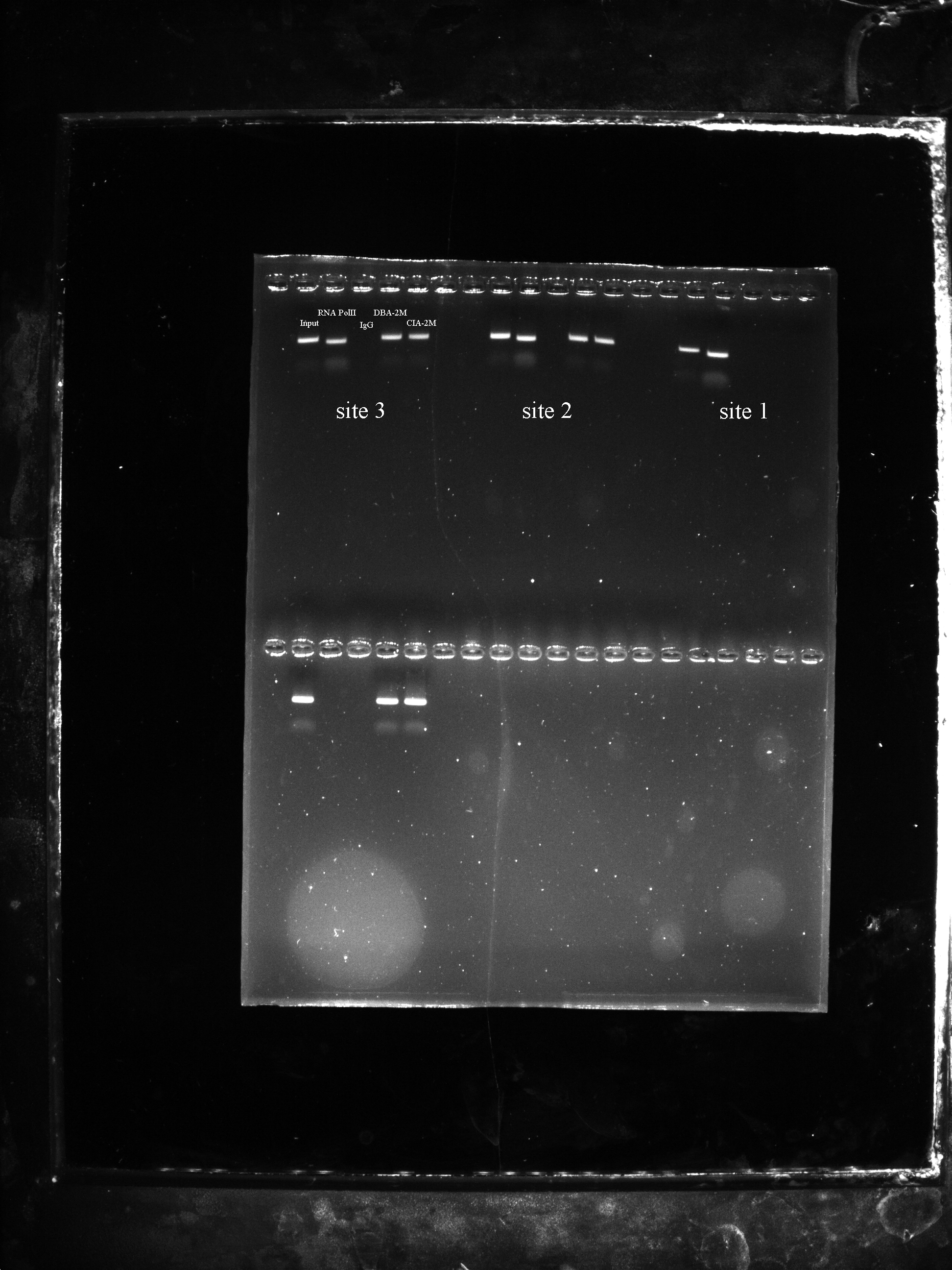

Supplement: Figure 6—source data 1. [file elife-85553-fig6-data1.zip › gel figure (labelled).tif]

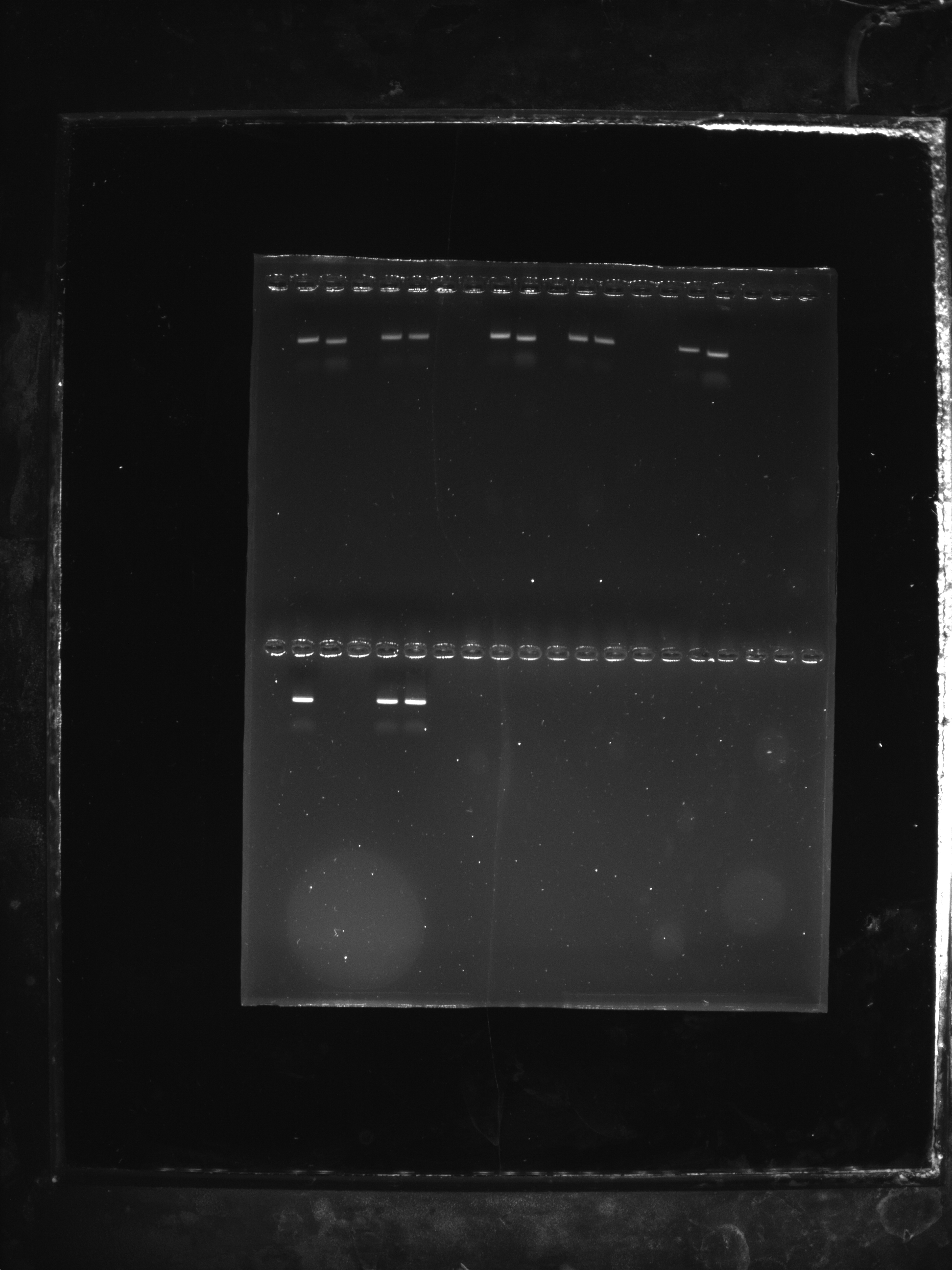

Supplement: Figure 6—source data 1. [file elife-85553-fig6-data1.zip › gel figure(raw).tif]
